# A novel adjuvant system BK-02 with CpG2006 and MF59 enhances the immunogenicity of a herpes zoster subunit vaccine

**DOI:** 10.3389/fimmu.2025.1641109

**Published:** 2025-07-15

**Authors:** Zihan Wang, Yingnan Guo, Xin Tang, Ying Sun, Tingting Wu, Hanyu Peng, Cenrong Wang, Weiheng Su, Chunlai Jiang, Yang Zang, Yaru Quan, Kangwei Xu, Bo Sun

**Affiliations:** ^1^ National Engineering Laboratory for AIDS Vaccine, School of Life Sciences, Jilin University, Changchun, China; ^2^ R&D Center, Changchun BCHT Biotechnology Co., Changchun, China; ^3^ Key Laboratory for Molecular Enzymology and Engineering, The Ministry of Education, School of Life Sciences, Jilin University, Changchun, China; ^4^ National Institutes for Food and Drug Control, National Health Commission (NHC) Key Laboratory of Research on Quality and Standardization of Biotech Products, National Medical Products Administration (NMPA) Key Laboratory for Quality Research and Evaluation of Biological Products, Beijing, China

**Keywords:** herpes zoster vaccine, glycoprotein E, adjuvant system, CpG ODN, MF59, cellular immunity

## Abstract

**Introduction:**

Reactivation of the varicella-zoster virus (VZV) results in herpes zoster (HZ), which can lead to complications such as postherpetic neuralgia. The commercially available HZ subunit adjuvanted vaccine, Shingrix®, offers significant protection against HZ in older adults. However, the adjuvant system of this vaccine has limitations that necessitate the development of alternative adjuvant systems.

**Methods:**

In this study, we established a novel adjuvant system, BK-02, composed of both the Toll-like receptor 9 (TLR9) agonist BK-02C (CpG2006) and a squalene-based oil-in-water emulsion, BK-02M (MF59), using ELISA, ELISpot, and flow cytometry analyses.

**Results:**

Our results showed that when combined with glycoprotein E (gE), the active ingredient of a recombinant HZ vaccine, the BK-02 adjuvant system elicited significantly higher gE-specific IFN-γ^+^ T-cell responses (486 SFU/10⁶ cells, 121-fold increase vs gE alone) and IgG antibody titers (Lg titers 5.2 vs 3.4 for gE alone). The optimal dose (5 μg gE + 30 μg BK-02C + 1× BK-02M) for inducing gE protein-specific cellular immunity was determined in mice. This corresponded to a clinical dose of “50 μg gE + 300/500 μg BK-02C + 0.5 mL BK-02M.” Additionally, pilot-scale samples of the recombinant HZ vaccine demonstrated enhanced gE-specific CD4^+^ and CD8^+^ T-cell immune responses, compared to Shingrix®. Moreover, the gE/BK-02 adjuvant system induced a Th1-regulated mixed immune response, enabling robust cellular and humoral immunity.

**Discussion:**

These findings indicated that the BK-02 adjuvant system is a promising adjuvant candidate for the current HZ subunit vaccines.

## 1 Introduction

Varicella zoster virus (VZV), a member of the genus *Varicellovirus* within the human Alphaherpesviruses, is a spherical virus of approximately 180–200 nanometers in diameter. VZV is a double-stranded DNA virus and has a genome of about 125 kb that encodes 71 genes and 67 proteins (1). Primary VZV infection results in varicella, following which the virus establishes latency within the dorsal root ganglia. The reactivation of latent VZV results in herpes zoster (HZ) (2). Although HZ can occur at any age groups in the populations, its reactivation is strongly linked to age-related decline in cell-mediated immunity (CMI), making it more common among older adults (3–5). While rarely life-threatening, a significant number of patients experience complications such as postherpetic neuralgia (PHN), which can last for extended periods and substantially affect their physical and psychological well-being (3, 6, 7). VZV-specific CMI is crucial for preventing HZ. Previous research indicated a strong correlation between diminished VZV-specific CMI and a higher incidence of HZ as well as increased rates of PHN (8–10). Conversely, high levels of VZV-specific antibodies provide no protection against HZ (8). Therefore, inducing a robust VZV-specific CMI through vaccination has become a central focus in the development of HZ vaccines.

Glycoprotein E (gE), the most abundantly expressed glycoprotein in VZV, plays a crucial role in mediating viral entry into host cells (11). Notably, gE contains both B cell and CD4^+^ T cell epitopes, enabling the induction of specific humoral and cellular immune responses. Because of its excellent immunogenicity, gE has been widely employed in the development of VZV vaccine development (12, 13). Currently, three HZ vaccines have been approved for clinical use, including Zostavax^®^ (Merck), Shingrix^®^ (GSK), and Live Vaccine (Changchun BCHT Biotechnology). Among these, the subunit vaccine Shingrix^®^ utilizes the recombinant gE as its antigen component, combined with the AS01B adjuvant system. Compared to the other two live-attenuated vaccines, Shingrix^®^ demonstrates significantly enhanced protection in elderly populations (14, 15). Clinical data show that Shingrix^®^ has achieved > 97.2% protection in individuals aged ≥ 50 years, with the protective efficacy remaining high (87%) even in those aged ≥ 80 years (16). Post-vaccination, both gE-specific CD4^+^ T cell immune responses and humoral immune responses are significantly higher than those induced by the live attenuated herpes zoster vaccine (17). Consequently, the FDA has recently recommended Shingrix^®^ as the preferred vaccine for HZ prevention. However, Shingrix^®^ is frequently associated with adverse reactions, including those localized to injection sites as well as systemic effects (14, 15). Moreover, its application has been limited by patent restrictions on the adjuvant, high production cost, and low manufacturing output. These limitations underscore the need to develop alternative adjuvant systems.

CpG oligodeoxynucleotides (ODNs) are immunomodulatory synthetic oligonucleotides specifically designed to stimulate the Toll-like receptor 9 (TLR9). Receptor activation triggers cellular signaling pathways that enhance both humoral and cellular immunity. ODNs are currently being developed as vaccine adjuvants for the prevention and treatment of cancers, infectious diseases, and allergies (18–22). Four distinct classes of CpG ODNs have been identified: Class B ODNs (also known as K-type), Class D ODNs (referred to as A-type), Class C ODNs, and Class P ODNs. Although each class contains at least one “CpG motif,” they differ in structure and immunological activity. Among them, Class B ODNs trigger pDC differentiation and TNFα production while stimulating B cell proliferation and IgM secretion, and they have been the most extensively characterized in clinical trials (18). ODN 2006 (also known as ODN 7909), a Class B CpG ODN with a high affinity for human TLR9, has been shown to elicit robust immune responses in both mice and humans, making it a promising candidate for vaccine adjuvant development (23–25). In addition, MF59, a squalene-based oil-in-water emulsion adjuvant, induces stronger humoral immune responses than traditional aluminum-based adjuvants. As the first non-aluminum adjuvant approved for human vaccines, MF59 demonstrates excellent tolerability with no significant safety concerns even in more “sensitive” populations (26, 27).

Recent reports indicated that combining both MF59 and CpG ODNs enhanced vaccine-induced T-cell responses (28, 29). Additionally, both MF59 and CpG adjuvants have been demonstrated to provide superior protective efficacy in elderly populations (30). MF59 has been extensively used as an adjuvant in seasonal influenza vaccines for older adults, with clinical studies confirming its significantly enhanced effectiveness compared to non-adjuvanted influenza vaccines (31). Even when administered in reduced-dose regimens, CpG-adjuvanted hepatitis B vaccines have shown better immunogenicity than traditional aluminum-adjuvanted vaccines in elderly recipients (32). Research on Shingrix^®^ has fully established the significant protective efficacy of its adjuvant system in older populations (14, 15). Our previous research demonstrated that the MF59/CpG2006 adjuvant system can induce immune responses comparable to those elicited by the Shingrix^®^ adjuvant system, suggesting that the MF59/CpG2006 combination holds substantial promise for clinical application and warrants further translational investigation (33).

In this study, we developed and evaluated a novel adjuvant system, designated BK-02, which is a combination of CpG2006 (BK-02C) and MF59 (BK-02M). Our major objective was to enhance the immunogenicity of the HZ gE subunit vaccine. Through systematic comparison with the commercially available Shingrix^®^ vaccine, we assessed the ability of the gE/BK-02 system to induce gE-specific cellular and humoral immune responses. Subsequent dose optimization and pilot-scale production further characterized the immunogenicity of the vaccine. Moreover, we observed that the gE/BK-02 adjuvant system induced a Th1-regulated mixed immune response, enabling robust cellular and humoral immunity. Our results demonstrate that the BK-02 adjuvant system significantly enhanced gE protein-specific cellular and humoral immunity, highlighting its potential as a promising alternative to the current HZ subunit vaccine adjuvants.

## 2 Materials and methods

### 2.1 Materials and reagents

The live attenuated HZ vaccine, MF59 (BK-02M) adjuvant, gE protein, and recombinant HZ vaccine produced via Chinese hamster ovary (CHO cell expression were provided by Changchun BCHT Biotechnology Co., Ltd. (Changchun, China). CpG2006 (BK-02C) adjuvant was purchased from Asymchem Laboratories Co., Ltd. (Tianjin, China). The recombinant HZ vaccine Shingrix^®^ was obtained from GSK plc (London, UK).

### 2.2 Animals and immunizations

Female C57BL/6J mice (3–4/6–8 weeks old) were purchased from Liaoning Changsheng Biotechnology Co., Ltd. (Liaoning, China) and divided into three experimental groups, i.e., A, B, and C (**Table 1**). In experiments A and C, mice were pre-immunized with 1000 plaque-forming units (PFU) of zoster-attenuated live vaccine on day 1, followed by primary and secondary immunizations on days 36 and 50, respectively. We employed a prime-boost immunization strategy with a 14-day interval between two doses of the recombinant HZ vaccine. The positive control group received Shingrix^®^, while the negative control was administered phosphate-buffered saline (PBS). Blood and spleen samples were collected on days 78 (experiment A) and 64 (experiment C). In experiment B, the mice received the same pre-immunization (1000 PFU on day 1), with primary and secondary immunizations administered on days 43 and 57, respectively. Blood and spleen samples were collected on day 71. Blood samples were obtained via orbital venous plexus puncture and collected in 1.5 mL EP tubes. Spleens were aseptically excised, with fascia and connective tissue carefully removed.

**Table 1:** Immunization and experimental groupings of animals in this study.

Experiment	Number of mice (n)	Treatment
A	5	5 μg gE/50 μg BK-02C + 1× BK-02M
	5	Shingrix^®^
	5	5 μg gE/1× BK-02M
	5	5 μg gE/50 μg BK-02C
	5	5 μg gE + PBS
	5	PBS
B	6	5 μg gE + 30 μg BK02C+1× BK02M
	6	10 μg gE + 30 μg BK02C +1× BK02M
	6	10 μg gE + 50 μg BK02C +1× BK02M
	6	5 μg gE + 50 μg BK02C +1× BK02M
	6	5 μg gE + 50 μg BK02C +2× BK02M
	6	5 μg gE + 30 μg BK02C +2× BK02M
	6	10 μg gE + 30 μg BK02C +2× BK02M
	6	10 μg gE + 50 μg BK02C +2× BK02M
	6	5 μg gE + 10 μg BK02C +1× BK02M
	6	5 μg gE + 70 μg BK02C +1× BK02M
	6	Shingrix^®^
	6	PBS
C	8	Pilot-scale sample 1
	8	Pilot-scale sample 2
	8	Pilot-scale sample 3
	8	Shingrix^®^
	8	PBS

Dosage equivalents: 1× BK-02M = 25 μL; 2× BK-02M = 50 μL. Shingrix^®^ dose is diluted to 10% of the original concentration.

### 2.3 Quantification of gE protein-specific IFN-γ-secreting T cells based on ELISpot assay

Splenocytes were isolated and adjusted to a working concentration of 5 × 10^6^ cells/mL. The specific procedures were as follows: a 70 μm cell strainer was placed in a culture dish, added with 2 mL of 1640 medium, and a single spleen tissue was placed on the strainer and ground using the plunger of a syringe. The ground cells were transferred to a 15 mL centrifuge tube for centrifugation at 400 g for 15 min at room temperature. The supernatant was discarded. A total of 2.5 mL of 1640 medium was added to resuspend the cells, then a total of 5 mL of ACK lysing buffer was added and incubated at room temperature for 5 min with occasional shaking, followed by centrifugation at 200 g for 10 min at room temperature. The supernatant was discarded, and the sample was resuspend in 10 mL of RPMI-1640 medium, and then centrifuged at 200 g for 5 min at room temperature. The supernatant was discarded and the sample was resuspend in 1 mL of mouse lymphocyte medium. The splenocytes was diluted 50-fold, then 20 μL of cells were mixed evenly with 20 μL of trypan blue (a 2-fold dilution of cells); finally, a total of 20 μL of the mixture was added to a hemocytometer and the total number of viable cells (transparent and unstained with a reflective halo) was counted in 4 squares. The cell density (cells/mL) = (total cell count in 4 squares/4) × 10^4^ × dilution factor × 2. The splenocytes were diluted to 5×10^6^ cells/mL using mouse lymphocyte medium for subsequent assays. The cell suspension (100 μL/well) was co-cultured with the gE protein stimulant (10 μg/mL, 100 μL/well) in ELISpot plates, which were incubated at 37°C with 5% CO_2_. Spot formation was detected according to the manufacturer’s instructions 24 h post-incubation.

### 2.4 Detection of gE protein-specific IgG antibodies and IgG subtypes based on ELISA

The 96-well plates were coated with gE protein (1 μg/mL, 50 μL/well) overnight at 4°C. Following washing three times and blocking, serial dilutions of mouse serum samples were prepared, starting at 1:10 dilution, with the highest dilution factor of 5 × 10^5^. After three washes with 0.05% PBS-T, HRP-conjugated goat anti-mouse IgG (Thermo Fisher, USA, Cat. no. A16078), HRP-conjugated goat anti-mouse IgG1 (Abcam, UK, Cat. no. Ab97240), HRP-conjugated goat anti-mouse IgG2b (Abcam, UK, Cat. no. Ab97250), and HRP-conjugated goat anti-mouse IgG2c (Abcam, Cat. no. Ab97255) were added to detect antigen-specific total IgG, IgG1, IgG2b, and IgG2c levels, respectively. The reaction was developed with the TMB substrate and terminated with sulfuric acid. The optical density (OD) was measured at a wavelength of 450 nm. Antibody titers were calculated based on the standard curves.

### 2.5 Th1/Th2 cytokine detection

Splenocytes were prepared at 5 × 10^6^ cells/mL and stimulated with gE protein in 96-well plates. After 46–48 h of incubation, the supernatants were collected and analyzed for IFN-γ, IL-2, IL-4, and IL-10 profiles using the Mouse Th1/Th2 Uncoated ELISA Kit (Invitrogen, USA, Cat. no. 88-7711-44) in accordance with the manufacturer’s protocol. The detailed steps were as follows: The capture antibodies were diluted to their working concentrations (IFN-γ 1:1000, IL-2 1:250, IL-4 1:250, and IL-10 1:250), then 100 µL was added per well and coated overnight at 4°C. After blocking, 100 µL of diluted supernatant samples was added per well, along with the standard working solution for in-plate gradient dilution. The plate was incubated at room temperature for 2 hours, then biotin-labeled detection antibodies (IFN-γ 1:1000, IL-2 1:250, IL-4 1:250, and IL-10 1:250) were added and incubated for 1 hour at room temperature. Next, 100 µL of streptavidin-HRP working solution (1:100 dilution) was added per well and incubated for 30 minutes at room temperature. Finally, TMB substrate was added for color development, the reaction was stopped after 15 minutes, and the absorbance was measured at 450 nm. The cytokine concentrations were determined using a standard curve.

### 2.6 Flow cytometric analysis of gE protein-specific CD4+/CD8+ T cell responses

Splenocytes were prepared at 1.2 × 10^7^ cells/mL (100 μL/well) and stimulated with gE protein (10 μg/mL, 50 μL/well) in 96-well plates. After 24 h of incubation, a GolgiPlug protein transport inhibitor was added to enhance cytokine accumulation. 50 μL 5× protein transport inhibitor cocktail was added to 96-well plates, and the plates were incubated overnight. The 96-well plates were then centrifuged at 600 g for 5 minutes at room temperature. Cells were washed once with 150 μl of cell staining buffer. Subsequently, cells were stained for surface markers. 200 μl fixation buffer was added to each well to fix the cells, ensuring complete resuspension of cells in the added solution. The plates were incubated at room temperature in the dark for 20–60 minutes. 200 μl 1× permeabilization buffer was added to each well, followed by centrifugation at 800 g for 5 minutes, after which the supernatant was discarded. The pellet was resuspended in the remaining volume, and the volume was adjusted to approximately 100 μl using 1× permeabilization buffer. Finally, cells were stained for intracellular cytokines prior to flow cytometric analysis.

### 2.7 Statistical analysis

Data were analyzed and graphically presented using the GraphPad Prism 10.4.1 (GraphPad Software, USA). Data in the figures were presented as geometric means with 95% confidence interval (CI), while data in the tables were presented as means ± standard error of mean (SEM). Statistical significance among multiple groups was determined by one-way analysis of variance (ANOVA), followed by t-tests for pairwise comparisons based on *P* < 0.05.

## 3 Results

### 3.1 gE/BK-02 adjuvant system induces gE protein-specific cellular and humoral immunity in immunized mice

**Figure 1:**
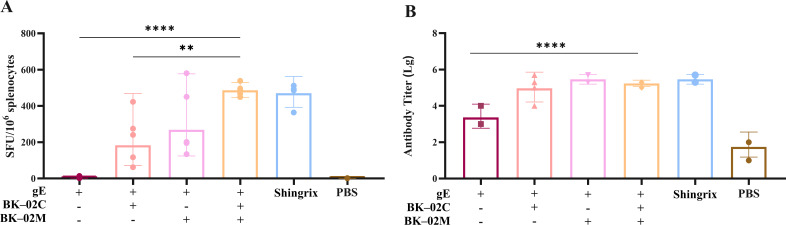
Evaluation of cellular and humoral immune responses induced by the gE/BK-02 adjuvant system in mice. **(A)** gE protein-specific IFN-γ-secreting T cells in splenocytes quantified by ELISpot. **(B)** gE-specific IgG antibody titers in serum measured by ELISA. **P < 0.01, ****P < 0.0001.

Our BK-02 adjuvant system consisted of both BK-02C (CpG2006) and BK-02M (MF59). To assess its effectiveness, we combined BK-02 with the active component (gE protein) of a recombinant HZ vaccine (produced via CHO cell expression) (experiment A in **Table 1**). Consistent with studies on Shingrix^®^, we also employed CMI as a key immunological endpoint, given its strong correlation with a reduced incidence of HZ and a lower likelihood of PHN development (8–10, 34). The number of gE protein-specific IFN-γ-secreting cells in mouse splenocytes was quantified by ELISpot (**Figure 1A**). Results were expressed as spot-forming unit (SFU) per 1 × 10^6^ cells. The gE, gE/BK-02C, gE/BK-02M, and “gE/BK-02 C + BK-02M” groups induced 4, 183, 268, and 486 SFUs, respectively. The “gE/BK-02C + BK-02M” group exhibited the highest IFN-γ response. Additionally, we measured gE-specific IgG antibody titers in the mouse serum (**Figure 1B**). After log transformation (Lg), the antibody titers were 3.4 (gE group), 5.0 (gE/BK-02C), 5.5 (gE/BK-02M), 5.2 (gE/BK-02C + BK-02M), 5.5 (Shingrix^®^), and 1.7 (PBS control). No significant differences were observed between adjuvant groups.

### 3.2 Th1/Th2 cytokine secretion

After stimulating mouse splenocytes with gE protein, we measured IFN-γ, IL-2, IL-4, and IL-10 levels induced by 5 μg gE/50 μg BK-02C + 1× BK-02M and Shingrix^®^ (**Table 2**). By measuring the levels of these cytokines, we determined the Th1/Th2 cytokine polarization induced in the experimental groups. The results showed that the gE/BK-02 adjuvant system induced relatively high levels of IFN-γ, IL-10, and IL-2, and low IL-4 levels, which were generally consistent with those induced by Shingrix^®^.

**Table 2:** Th1/Th2 cytokine secretion profile.

Group	IFN-γ	IL-2	IL-4	IL-10
5 μg gE/50 μg BK-02C +1× BK-02M	9187.7 ± 2578	607.2 ± 145.8	23.6 ± 2.56	544.9 ± 185.4
Shingrix^®^	10892.4 ± 3896	400.6 ± 60.14	27.4 ± 1.53	549.3 ± 89.7

Data are presented as means ± SEM. No significant difference is detected between 5 μg gE/50 μg BK-02C + 1× BK-02M and Shingrix^®^ treatments.

### 3.3 Optimization of gE/BK-02 adjuvant system formulation

**Figure 2:**
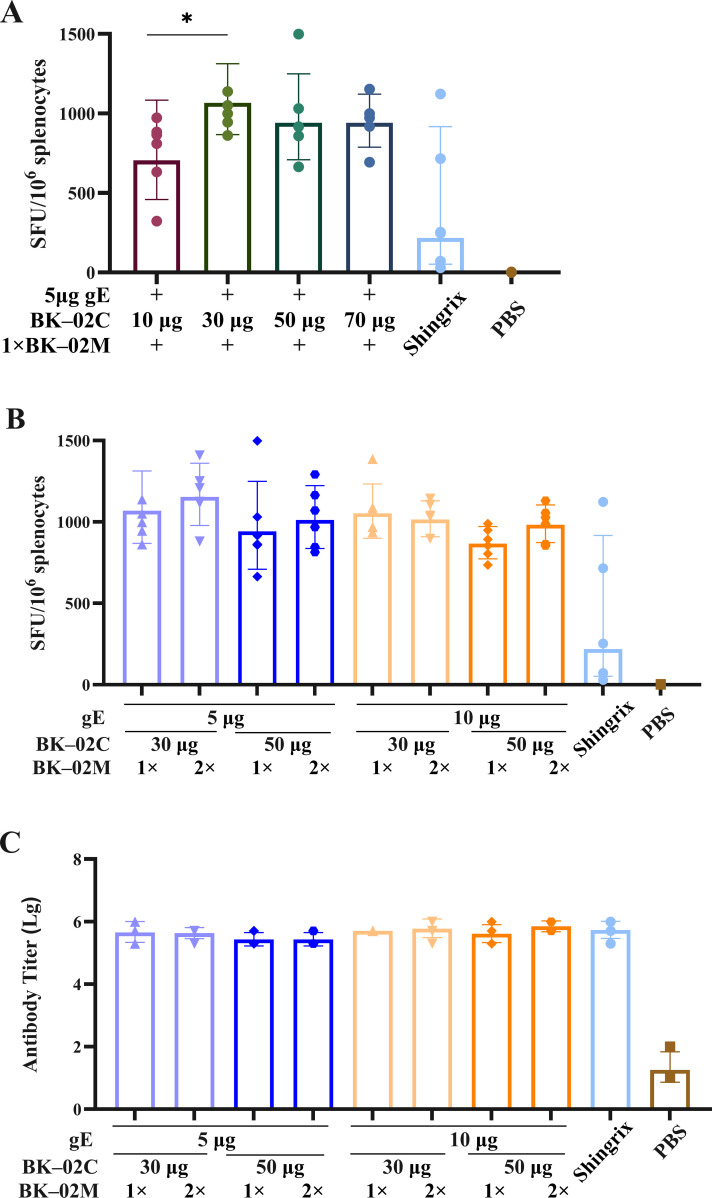
Evaluation of cellular and humoral immune responses induced by different gE/BK-02 adjuvant formulations in mice. **(A)** Quantification of gE-specific IFN-γ-secreting T cells in splenocytes based on ELISpot after immunization with gE protein combined with 10, 30, 50, or “70 μg BK-02C + 1× BK-02M.” **(B)** gE-specific IFN-γ^+^ T cell responses (ELISpot) elicited by varying combinations of gE protein (5/10 μg), BK-02C (30/50 μg), and BK-02M (1×/2×). **(C)** gE-specific IgG antibody titers in serum measured by ELISA. *P < 0.05.

To determine the optimal dose ratio of the active component (gE protein) of the recombinant HZ vaccine and the BK-02 adjuvant system, we conducted a series of immunization experiments (experiment B in **Table 1**). Initial screening of the BK-02C adjuvant doses (10, 30, 50, and 70 μg) revealed that 30 μg BK-02C induced the highest number of gE-specific IFN-γ SFU (1068 SFU/10^6^ cells), followed by 50 μg (942 SFUs), 70 μg (940 SFUs), and 10 μg (705 SFUs) (**Figure 2A**). Based on these results, 30 μg and 50 μg BK-02C were selected for further evaluation.

Considering that Shingrix^®^ used 50–100 μg gE in clinical studies (with mouse doses typically one-tenth of human doses), we evaluated 5 μg and 10 μg gE. Both doses induced comparable IFN-γ responses (1068 vs. 1053 SFUs for 30 μg BK-02C; 942 vs. 867 SFUs for 50 μg BK-02C), with slightly higher responses observed at 5 μg (**Figure 2B**). This finding was consistent with the pharmacodynamic data derived from Shingrix^®^ studies in mice, establishing 5 μg gE as the optimal dose.

Comparative analysis of 30 μg vs. 50 μg BK-02C (with fixed 5 μg gE and 1× BK-02M) showed marginally stronger responses with 30 μg (1068 SFUs for 30 μg vs. 942 SFUs for 50 μg). For BK-02M, no significant difference was observed between the 1× and 2× doses (1068 vs. 1153 SFUs), leading to the selection of the standard 1× dose (equivalent to the MF59 content in licensed influenza vaccines). All formulations induced high gE-specific IgG titers without significant differences (**Figure 2C**).

### 3.4 Cellular immune responses induced by pilot-scale recombinant HZ vaccine

**Figure 3:**
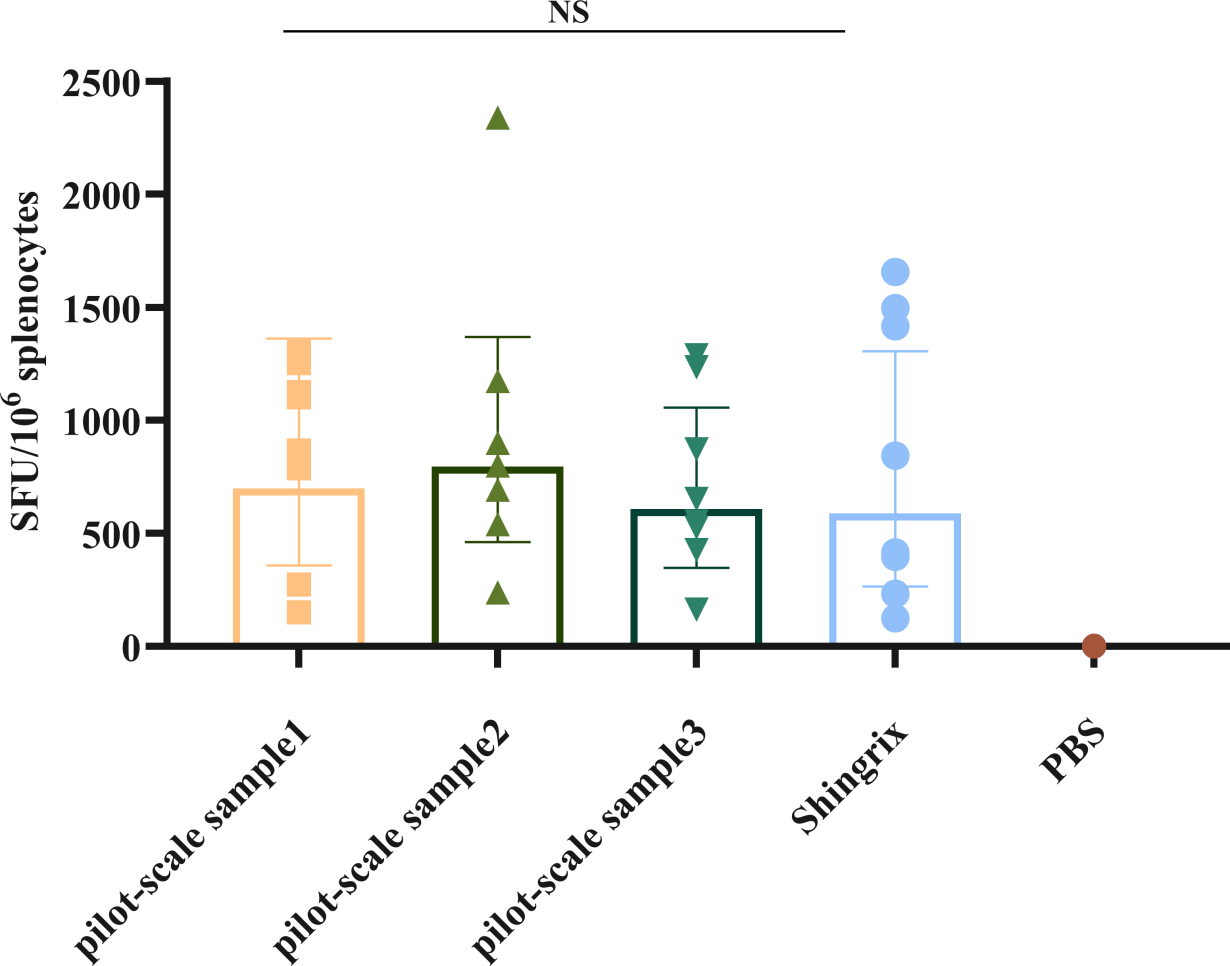
gE-specific IFN-γ-secreting T cells induced by a pilot-scale recombinant HZ vaccine produced via Chinese hamster ovary (CHO) cell expression. NS, not significant.

Three pilot-scale production batches of the recombinant HZ vaccine, produced via CHO cell expression (Lots P1, P2, and P3), were formulated with “50 μg gE/300 μg BK-02C + 0.5 mL BK-02M” (experiment C in **Table 1**). All three pilot-scale batches induced robust cellular immunity, with gE-specific IFN-γ SFU per 1 × 10^6^ cells measured at 700, 795, and 607 SFUs for the recombinant vaccine batches vs. 589 SFUs for Shingrix^®^ (**Figure 3**). No significant inter-batch variations compared to Shingrix^®^ treatment were observed.

**Figure 4:**
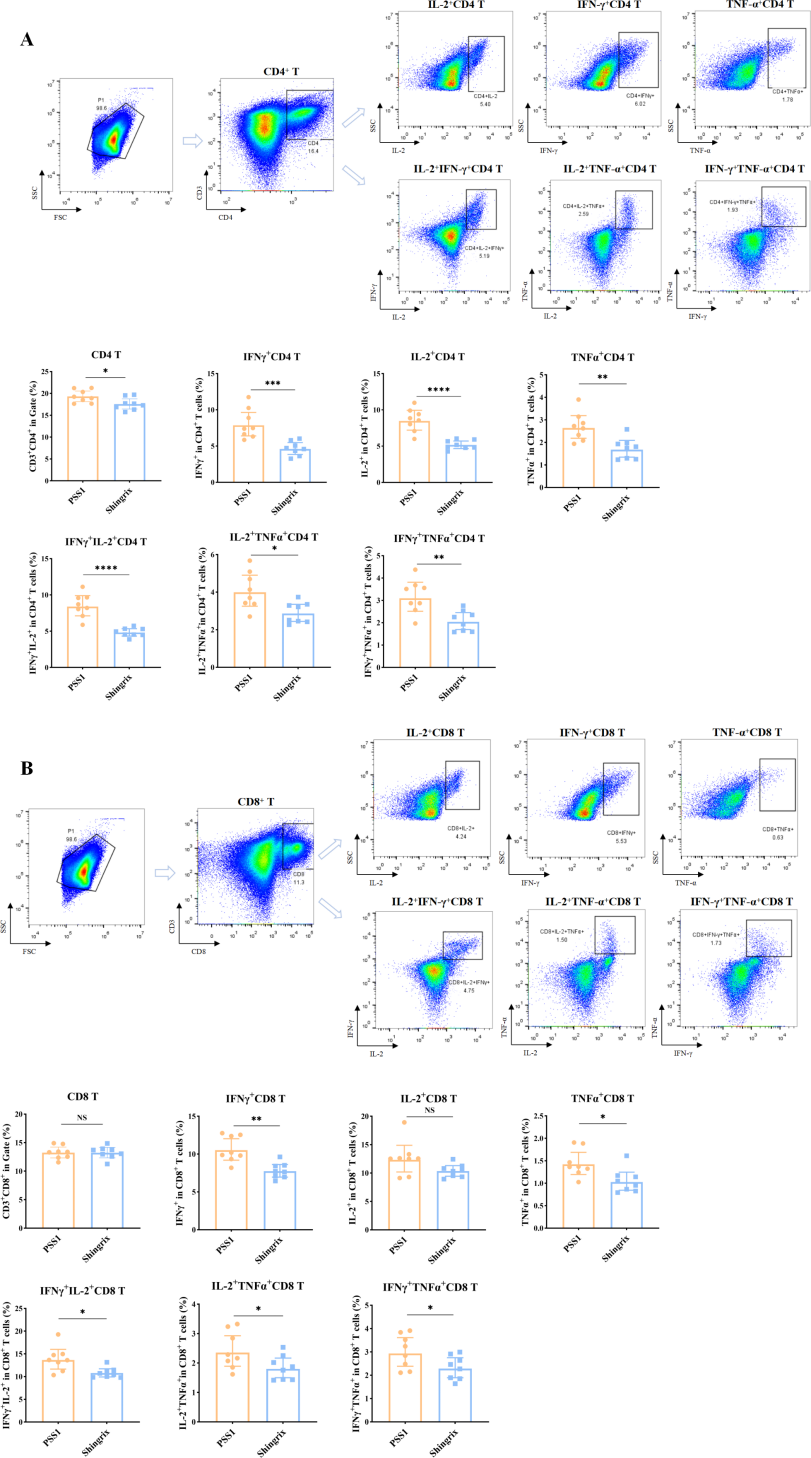
Cellular immune responses induced by a pilot-scale recombinant HZ vaccine produced via Chinese hamster ovary (CHO) cell expression. **(A)** Flow cytometric analysis of cytokine-positive percentages in CD4^+^ T cells (IFN-γ^+^, IL-2^+^, and TNF-α^+^ single/double-positive subsets), showing the images of dot plots and gating strategy as well as the corresponding statistical analyses. **(B)** Flow cytometric analysis of cytokine-positive percentages in CD8^+^ T cells (IFN-γ^+^, IL-2^+^, and TNF-α^+^ single/double-positive subsets), showing the images of dot plots and gating strategy as well as the corresponding statistical analyses. The first plot in parts A and B shows the gated T cell population. *P < 0.05, **P < 0.01, ***P < 0.001, ****P < 0.0001; NS, not significant.

In addition, we examined the gE-specific CD4^+^ T/CD8^+^ T cell immune responses. Flow cytometry results showed that the pilot-scale sample 1 (PSS1) induced significantly higher numbers of CD4^+^ T cells, including gE protein-specific IFN-γ^+^, IL-2^+^, and TNF-α^+^ single-positive CD4^+^ T cells, as well as IL-2^+^IFN-γ^+^, IL-2^+^TNF-α^+^, and IFN-γ^+^TNF-α^+^ double-positive CD4^+^ T cells, compared to those for the Shingrix^®^ vaccine (**Figure 4A**). While no statistically significant difference was observed in total CD8^+^ T cell count compared to those of the Shingrix^®^ vaccine, PSS1 induced a significantly increase in the numbers of gE protein-specific IFN-γ^+^ and TNF-α^+^ single-positive CD8^+^ T cells, as well as IL-2^+^IFN-γ^+^, IL-2^+^TNF-α^+^, and IFN-γ^+^TNF-α^+^ double-positive CD8^+^ T cells in stimulated splenocytes from immunized mice (**Figure 4B**).

### 3.5 IgG antibody subtypes induced by pilot-scale recombinant HZ vaccine

**Figure 5:**
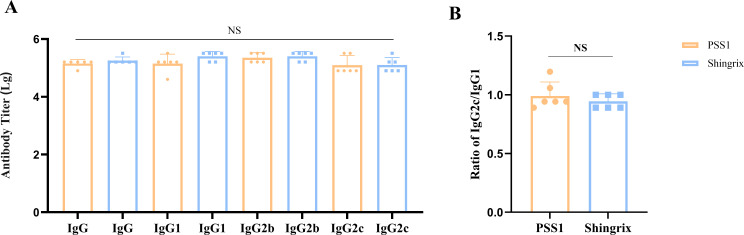
IgG antibody subtypes. **(A)** Antibody titers of IgG, IgG1, IgG2b, and IgG2c. **(B)** Ratio of IgG2c/IgG1. NS, not significant.

Our results demonstrated that PSS1 induced antibody titers comparable to those of Shingrix^®^, including total IgG, IgG1, IgG2b, and IgG2c, with no significant differences among the induced IgG subtypes (**Figure 5A**). To visually demonstrate Th1/Th2 polarization, we compared IgG2c and IgG1 levels using the IgG2c/IgG1 ratio (**Figure 5B**). Results showed that both PSS1 (0.99) and Shingrix^®^ (0.94) exhibited ratios slightly below 1 (IgG2c/IgG1 < 1), indicating marginally higher IgG1 levels. Notably, our pilot samples demonstrated a higher IgG2c/IgG1 ratio than Shingrix^®^, suggesting enhanced Th1-regulated mixed immune responses induced by our BK-02 adjuvant system.

## 4 Discussion

The reactivation of VZV results in HZ, posing a significant public health challenge. Its reactivation is strongly linked to age-related decline in CMI, making it more common among older adults (3–5). Among HZ vaccines currently approved for clinical use, Shingrix^®^ demonstrates the highest protective efficacy in elderly populations (14, 15). However, its adjuvant system has inherent limitations, highlighting the need to develop alternative adjuvant systems. In this study, we developed and evaluated a novel adjuvant system BK-02, which is a combination of CpG2006 (BK-02C) and MF59 (BK-02M).

### 4.1 Enhanced cellular and humoral immune responses by the gE/BK-02 adjuvant system

We combined BK-02 with the active component (gE protein) of a recombinant HZ vaccine. The “gE/BK-02C + BK-02M” group exhibited the highest IFN-γ response, indicating that the combined adjuvant system significantly enhanced gE-specific T-cell immunity compared to the gE group or single-adjuvant formulations. These results align with previous studies demonstrating that combinatorial adjuvants (e.g., AS01B in Shingrix^®^) enhance cellular immune responses by activating multiple innate immune pathways (35, 36). Notably, the “gE/BK-02C + BK-02M” group elicited a comparable IFN-γ response to the positive control (Shingrix^®^). Also, the adjuvanted gE formulations effectively induced robust humoral immunity. We observed that the use of MF59 significantly increased gE-specific IgG antibody titers, which is consistent with previous studies reporting that MF59 enhances humoral immune responses (37–39). In conclusion, the gE/BK-02 adjuvant system significantly enhances gE-specific cellular (IFN-γ^+^ T cells) and humoral (IgG) immune responses in mice. The combined effects of BK-02C and BK-02M effectively improved the immunogenicity of the HZ subunit vaccine. Given the crucial role of CMI in restricting VZV reactivation (8–10, 34), our adjuvant system has demonstrated its significant potential as an alternative adjuvant strategy.

### 4.2 Th1-regulated mixed immune response induced by gE/BK-02

When stimulated with different antigens, most CD4+ T cell lines differentiate into two functional subsets, Th1 and Th2, which produce distinct lymphokines and exhibit different effector functions, activation requirements, and cytoplasmic signaling mechanisms. Th1 cells primarily secrete IFN-γ, IL-2, and TNF-α, mediating cellular immunity with IFN-γ as the major marker. Th2 cells mainly produce IL-4, IL-5, IL-6, IL-10, and IL-13, mediating humoral immunity with IL-4 as the primary marker (40, 41). To systematically evaluate the immune polarization profiles induced by different adjuvanted gE vaccine formulations, we performed Th1/Th2 cytokine analysis. The results indicated that our gE/BK-02 adjuvant system induced a Th1-regulated mixed immune response, characterized by strong Th1-type cellular immunity (high level of IFN-γ), regulatory modulation (high level of IL-10), and Th2-type suppression (low level of IL-4). This pattern may be related to the composition of the adjuvant systems. Previous studies have demonstrated that CpG activates dendritic cells (DCs) and B cells through TLR9 to promote IFN-γ secretion and induce Th1-type immune responses (42). CpG ODN may also activate B cells to secrete IL-10 via TLR9 (43). MF59 itself enhances antigen uptake by activating DCs and promotes Th2-biased immune responses; however, when combined with CpG, it induces Th1-type immune responses (44). In summary, the gE/BK-02 adjuvant system induced a Th1-regulated mixed immune response, demonstrating its ability to induce cellular immune responses and mediate immunoregulation. The Th1/Th2 cytokine profile was comparable to that of the Shingrix^®^, consistent with our previous findings (33).

### 4.3 Optimal dose and pilot-scale validation of the gE/BK-02 formulation

The dose ratio study of the active ingredient (gE protein) of the recombinant HZ vaccine and the BK-02 adjuvant system identified the optimal dose group for mouse studies as “5 μg gE/30 μg BK-02C + 1× BK-02M.” This is consistent with prior preclinical studies of gE-based vaccines, showing that 5 μg gE combined with adjuvants (e.g., AS01B) elicited maximal T-cell responses in mice (34). Calculated via a 10-fold extrapolation from the murine dose, the clinical doses were established as “50 μg gE/300 μg BK-02C + 0.5 mL BK-02M” and “50 μg gE/500 μg BK-02C + 0.5 ml BK-02M” for the standard and high dose groups, respectively. This scaling approach is consistent with FDA guidelines for vaccine dose translation. Notably, Shingrix^®^ uses 50 μg gE clinically (45), supporting the feasibility of our extrapolated dose, while the higher adjuvant dose (500 μg BK-02C) may provide superior therapeutic efficacy (46). In conclusion, “5 μg gE/30 μg BK-02C + 1×BK-02M” represents the optimal dose for inducing gE protein-specific cellular immunity in mice.

After successfully formulating the gE recombinant HZ vaccine, produced via CHO cell expression and containing the BK-02 adjuvant system at the pilot scale, we found that the pilot-scale sample induced superior cellular immune responses compared to those induced by the Shingrix^®^ vaccine in immunized mice. All three pilot-scale batches of the recombinant HZ vaccine induced gE protein-specific T-cell responses with no significant inter-batch variations, consistent with the high reproducibility of industrial-scale CHO cell cultures (47). The pilot-scale recombinant HZ vaccine demonstrated improved gE protein-specific CD4^+^ T and CD8^+^ T cell immune responses compared with the Shingrix^®^ vaccine. While Shingrix^®^ relies on liposome-based delivery of AS01B (35), our BK-02 system may more effectively cross-present antigens through the proposed DC activation pathway of BK-02 (42, 48).

Finally, we examined the IgG antibody subtypes induced by the pilot-scale samples in mouse models to validate our Th1/Th2 cytokine secretion analysis. Human IgG is divided into four subtypes (IgG1 to IgG4), whereas mouse IgG has five subtypes (IgG1, IgG2a, IgG2b, IgG2c, and IgG3). Notably, human IgG1 and IgG3 strongly correlate with Th1 responses, while IgG2 and IgG4 associate with Th2 responses. In mice, IgG1 is strongly linked to Th2, both IgG2a and IgG2c to Th1, and IgG2b to both Th1 and Th2 cells (49–52). Our Th1/Th2 cytokine secretion experiments demonstrated that the gE/BK-02 adjuvant system elicited a Th1-regulated mixed immune response, which was further confirmed by the IgG subtype analysis, indicating that our BK-02 adjuvant system has potent humoral immunostimulatory capacity. CpG2006 directly activates Th1 via the TLR9 pathway. This activation increases IFN-γ levels and elevates the levels of IgG2b/2c, while indirectly inducing IL-10 secretion in B cells (42, 43). IL-10 secretion enhances IgG1 production, which helps achieve a balanced IgG2c/IgG1 ratio, approaching 1. Meanwhile, MF59 enhances antigen presentation to maintain total IgG levels and synergizes with CpG to suppress Th2 responses, leading to reduced IL-4 production (44). In summary, our gE/BK-02 adjuvant system induces a Th1-regulated mixed immune response, activating both cellular and humoral immunity. Moreover, it generates IgG antibody profiles comparable to that of Shingrix^®^, demonstrating its potential as a promising adjuvant candidate for next-generation HZ subunit vaccines.

### 4.4 Limitations

Several limitations of our study are noted. Given that HZ predominantly occurs in elderly populations with compromised CMI (3–5), subsequent experiments should incorporate aged mouse models. Furthermore, due to the strict species specificity of VZV, which exclusively causes clinical symptoms in humans and non-human primates, murine models cannot support viral latency or reactivation (53). Therefore, comprehensive efficacy evaluation in rhesus macaque models represents an essential approach for preclinical development. Moreover, we did not evaluate neutralizing antibody responses, which represent an important functional component of vaccine-induced immunity (54). Subsequent work should include neutralization assays to more comprehensively assess the protective capacity of antibodies induced by the BK-02 adjuvant system. Addressing these aspects will provide a more complete understanding of our vaccine candidate’s potential.

## 5 Conclusions

In this study, we developed a novel adjuvant system, BK-02, consisting of both BK-02C (CpG2006) and BK-02M (MF59). We systematically evaluated the immunogenicity of the BK-02 adjuvant system in combination with the recombinant varicella-zoster virus glycoprotein E (gE) in mice. Our results demonstrated that the gE/BK-02 adjuvant system effectively induced robust cellular and humoral immune responses comparable to Shingrix^®^, with optimal immunogenicity achieved at a dose of “5 μg gE + 30 μg BK-02C + 1× BK-02M” in mice. The adjuvant system’s efficacy was further confirmed in pilot-scale vaccine production. These effects are attributed to the combined action of CpG2006-mediated Th1 activation and MF59-enhanced antigen presentation. This study establishes BK-02 as a promising alternative adjuvant for HZ vaccines, with several critical next steps. Comprehensive evaluation in aged animal models to better recapitulate the immunosenescent state of elderly populations. Comprehensive efficacy evaluation in rhesus macaque models prior to clinical trials. Phase I clinical trials to assess safety and immunogenicity in human subjects.

## Data Availability

The original contributions presented in the study are included in the article/supplementary material. Further inquiries can be directed to the corresponding authors.

## References

[1] Zerboni L Sen N Oliver SL Arvin AM. Molecular mechanisms of varicella zoster virus pathogenesis. Nat Rev Microbiol. (2014) 12:197–210. doi: 10.1038/nrmicro3215, PMID: 24509782 PMC4066823

[2] Arvin AM. Varicella-zoster virus: molecular virology and virus-host interactions. Curr Opin Microbiol. (2001) 4:442–9. doi: 10.1016/S1369-5274(00)00233-2, PMID: 11495809

[3] Johnson RW. Herpes zoster and postherpetic neuralgia. Expert Rev Vaccines. (2010) 9:21–6. doi: 10.1586/erv.10.30, PMID: 20192714

[4] John AR Canaday DH. Herpes zoster in the older adult. Infect Dis Clin North Am. (2017) 31:811–26. doi: 10.1016/j.idc.2017.07.016, PMID: 29079160 PMC5724974

[5] Varghese L Standaert B Olivieri A Curran D. The temporal impact of aging on the burden of herpes zoster. BMC Geriatr. (2017) 17:30. doi: 10.1186/s12877-017-0420-9, PMID: 28114907 PMC5259900

[6] Ishihara R Watanabe R Shiomi M Katsushima M Fukumoto K Yamada S. Exploring the link between varicella-zoster virus, autoimmune diseases, and the role of recombinant zoster vaccine. Biomolecules. (2024) 14:739. doi: 10.3390/biom14070739, PMID: 39062454 PMC11274381

[7] Tyring SK. Management of herpes zoster and postherpetic neuralgia. J Am Acad Dermatol. (2007) 57:S136–42. doi: 10.1016/j.jaad.2007.09.016, PMID: 18021865

[8] Asada H. VZV-specific cell-mediated immunity, but not humoral immunity, correlates inversely with the incidence of herpes zoster and the severity of skin symptoms and zoster-associated pain: The SHEZ study. Vaccine. (2019) 37:6776–81. doi: 10.1016/j.vaccine.2019.09.031, PMID: 31543415

[9] Shirane R Tang H Hayashi K Okuno Y Iso H Asada H. group, S. s., Relationship between cell-mediated immunity to Varicella-Zoster virus and aging in subjects from the community-based Shozu Herpes Zoster study. J Med Virol. (2017) 89:313–7. doi: 10.1002/jmv.24629, PMID: 27420414

[10] Steain M Sutherland JP Rodriguez M Cunningham AL Slobedman B Abendroth A. Analysis of T cell responses during active varicella-zoster virus reactivation in human ganglia. J Virol. (2014) 88:2704–16. doi: 10.1128/JVI.03445-13, PMID: 24352459 PMC3958057

[11] Grose C. Glycoproteins encoded by varicella-zoster virus: biosynthesis, phosphorylation, and intracellular trafficking. Annu Rev Microbiol. (1990) 44:59–80. doi: 10.1146/annurev.mi.44.100190.000423, PMID: 2174668

[12] Wu J Li H Yuan Y Wang R Shi T Li Z. Truncated VZV gE induces high-titer neutralizing antibodies in mice. Vaccines (Basel). (2024) 12:1139. doi: 10.3390/vaccines12101139, PMID: 39460306 PMC11510871

[13] Liu J Lin J Cai L Sun J Ding X Wang C. Immunogenicity of varicella zoster virus DNA vaccines encoding glycoprotein E and immediate early protein 63 in mice. Viruses. (2022) 14 :1214. doi: 10.3390/v14061214, PMID: 35746685 PMC9230688

[14] James SF Chahine EB Sucher AJ Hanna C. Shingrix: the new adjuvanted recombinant herpes zoster vaccine. Ann Pharmacother. (2018) 52:673–80. doi: 10.1177/1060028018758431, PMID: 29457489

[15] Tricco AC Zarin W Cardoso R Veroniki AA Khan PA Nincic V. Efficacy, effectiveness, and safety of herpes zoster vaccines in adults aged 50 and older: systematic review and network meta-analysis. BMJ. (2018) 363:k4029. doi: 10.1136/bmj.k4029, PMID: 30361202 PMC6201212

[16] Chlibek R Pauksens K Rombo L van Rijckevorsel G Richardus JH Plassmann G. Long-term immunogenicity and safety of an investigational herpes zoster subunit vaccine in older adults. Vaccine. (2016) 34:863–8. doi: 10.1016/j.vaccine.2015.09.073, PMID: 26432913

[17] Leroux-Roels I Leroux-Roels G Clement F Vandepapeliere P Vassilev V Ledent E. A phase 1/2 clinical trial evaluating safety and immunogenicity of a varicella zoster glycoprotein e subunit vaccine candidate in young and older adults. J Infect Dis. (2012) 206:1280–90. doi: 10.1093/infdis/jis497, PMID: 22872734

[18] Kayraklioglu N Horuluoglu B Klinman DM. CpG oligonucleotides as vaccine adjuvants. Methods Mol Biol. (2021) 2197:51–85. doi: 10.1007/978-1-0716-0872-2_4, PMID: 32827132

[19] Wilson HL Dar A Napper SK Marianela Lopez A Babiuk LA Mutwiri GK. Immune mechanisms and therapeutic potential of CpG oligodeoxynucleotides. Int Rev Immunol. (2006) 25:183–213. doi: 10.1080/08830180600785868, PMID: 16818371

[20] Verthelyi D Kenney RT Seder RA Gam AA Friedag B Klinman DM. CpG oligodeoxynucleotides as vaccine adjuvants in primates. J Immunol. (2002) 168:1659–63. doi: 10.4049/jimmunol.168.4.1659, PMID: 11823494

[21] Klinman DM Klaschik S Sato T Tross D. CpG oligonucleotides as adjuvants for vaccines targeting infectious diseases. Adv Drug Delivery Rev. (2009) 61:248–55. doi: 10.1016/j.addr.2008.12.012, PMID: 19272313

[22] Hanagata N. CpG oligodeoxynucleotide nanomedicines for the prophylaxis or treatment of cancers, infectious diseases, and allergies. Int J Nanomedicine. (2017) 12:515–31. doi: 10.2147/IJN.S114477, PMID: 28144136 PMC5248940

[23] Liu Y Zhang Z Sang Q Zhang Y Jiang M Zhou H. Recombinant MUC1-MBP fusion protein combined with CpG2006 vaccine induces antigen-specific CTL responses through cDC1-mediated cross-priming mainly regulated by type I IFN signaling in mice. Immunol Lett. (2022) 245:38–50. doi: 10.1016/j.imlet.2022.04.002, PMID: 35405170

[24] Li N Zhang L Zheng B Li W Liu J Zhang H. RSV recombinant candidate vaccine G1F/M2 with CpG as an adjuvant prevents vaccine-associated lung inflammation, which may be associated with the appropriate types of immune memory in spleens and lungs. Hum Vaccin Immunother. (2019) 15:2684–94. doi: 10.1080/21645515.2019.1596710, PMID: 31021703 PMC6930060

[25] Valencia-Hernandez AM Zillinger T Ge Z Tan PS Cozijnsen A. G, I. M.; Lahoud, M. H.; Caminschi, I.; Barchet, W.; Heath, W. R.; Fernandez-Ruiz, D., Complexing CpG adjuvants with cationic liposomes enhances vaccine-induced formation of liver T(RM) cells. Vaccine. (2023) 41:1094–107. doi: 10.1016/j.vaccine.2022.12.047, PMID: 36609029

[26] O’Hagan DT Ott GS Nest GV Rappuoli R Giudice GD. The history of MF59((R)) adjuvant: a phoenix that arose from the ashes. Expert Rev Vaccines. (2013) 12:13–30. doi: 10.1586/erv.12.140, PMID: 23256736

[27] Zhang W Cui H Xu J Shi M Bian L Cui L. Biodistribution and mechanisms of action of MF59 and MF59-like adjuvants. J Control Release. (2025) 378:573–87. doi: 10.1016/j.jconrel.2024.12.044, PMID: 39719213

[28] Wang S Yang G Nie J Yang R Du M Su J. Recombinant E(rns)-E2 protein vaccine formulated with MF59 and CPG-ODN promotes T cell immunity against bovine viral diarrhea virus infection. Vaccine. (2020) 38:3881–91. doi: 10.1016/j.vaccine.2020.03.020, PMID: 32280039

[29] Yang J Hu X Chen X Li W Yin Q Xiong Y. A novel MF59 and CpG1018 adjuvant combination enhances the humoral and cellular immune responses against a truncated varicella-zoster viral glycoprotein E. Immunol Lett. (2025) 275:107025. doi: 10.1016/j.imlet.2025.107025, PMID: 40239819

[30] Nanishi E Angelidou A Rotman C Dowling DJ Levy O Ozonoff A. Precision vaccine adjuvants for older adults: A scoping review. Clin Infect Dis. (2022) 75:S72–80. doi: 10.1093/cid/ciac302, PMID: 35439286 PMC9376277

[31] Tsai TF. Fluad(R)-MF59(R)-adjuvanted influenza vaccine in older adults. Infect Chemother. (2013) 45:159–74. doi: 10.3947/ic.2013.45.2.159, PMID: 24265964 PMC3780956

[32] Janssen JM Jackson S Heyward WL Janssen RS. Immunogenicity of an investigational hepatitis B vaccine with a toll-like receptor 9 agonist adjuvant (HBsAg-1018) compared with a licensed hepatitis B vaccine in subpopulations of healthy adults 18–70 years of age. Vaccine. (2015) 33:3614–8. doi: 10.1016/j.vaccine.2015.05.070, PMID: 26067185

[33] He L Sun B Guo Y Yan K Liu D Zang Y. Immune response of C57BL/6J mice to herpes zoster subunit vaccines formulated with nanoemulsion-based and liposome-based adjuvants. Int Immunopharmacol. (2021) 101:108216. doi: 10.1016/j.intimp.2021.108216, PMID: 34634689

[34] Dendouga N Fochesato M Lockman L Mossman S Giannini SL. Cell-mediated immune responses to a varicella-zoster virus glycoprotein E vaccine using both a TLR agonist and QS21 in mice. Vaccine. (2012) 30:3126–35. doi: 10.1016/j.vaccine.2012.01.088, PMID: 22326899

[35] Didierlaurent AM Laupeze B Di Pasquale A Hergli N Collignon C Garcon N. Adjuvant system AS01: helping to overcome the challenges of modern vaccines. Expert Rev Vaccines. (2017) 16:55–63. doi: 10.1080/14760584.2016.1213632, PMID: 27448771

[36] Roman F Burny W Ceregido MA Laupeze B Temmerman ST Warter L. Adjuvant system AS01: from mode of action to effective vaccines. Expert Rev Vaccines. (2024) 23:715–29. doi: 10.1080/14760584.2024.2382725, PMID: 39042099

[37] Khurana S Verma N Yewdell JW Hilbert AK Castellino F Lattanzi M. MF59 adjuvant enhances diversity and affinity of antibody-mediated immune response to pandemic influenza vaccines. Sci Transl Med. (2011) 3:85ra48. doi: 10.1126/scitranslmed.3002336, PMID: 21632986 PMC3501657

[38] Khurana S Chearwae W Castellino F Manischewitz J King LR Honorkiewicz A. Vaccines with MF59 adjuvant expand the antibody repertoire to target protective sites of pandemic avian H5N1 influenza virus. Sci Transl Med. (2010) 2:15ra5. doi: 10.1126/scitranslmed.3000624, PMID: 20371470

[39] Li APY Cohen CA Leung NHL Fang VJ Gangappa S Sambhara S. Immunogenicity of standard, high-dose, MF59-adjuvanted, and recombinant-HA seasonal influenza vaccination in older adults. NPJ Vaccines. (2021) 6:25. doi: 10.1038/s41541-021-00289-5, PMID: 33594050 PMC7886864

[40] Mosmann TR Coffman RL. TH1 and TH2 cells: different patterns of lymphokine secretion lead to different functional properties. Annu Rev Immunol. (1989) 7:145–73. doi: 10.1146/annurev.iy.07.040189.001045, PMID: 2523712

[41] Kawakami K Parker DC. Differences between T helper cell type I (Th1) and Th2 cell lines in signalling pathways for induction of contact-dependent T cell help. Eur J Immunol. (1992) 22:85–93. doi: 10.1002/eji.1830220114, PMID: 1309700

[42] Krieg AM. CpG motifs in bacterial DNA and their immune effects. Annu Rev Immunol. (2002) 20:709–60. doi: 10.1146/annurev.immunol.20.100301.064842, PMID: 11861616

[43] Liu QB Zhou RH Liu CM. TLR9/FCRL3 regulates B cell viability, apoptosis, and antibody and IL-10 production through ERK1/2, p38, and STAT3 signaling pathways. In Vitro Cell Dev Biol Anim. (2022) 58:702–11. doi: 10.1007/s11626-022-00720-8, PMID: 36121575

[44] Yang M Yan Y Fang M Wan M Wu X Zhang X. MF59 formulated with CpG ODN as a potent adjuvant of recombinant HSP65-MUC1 for inducing anti-MUC1+ tumor immunity in mice. Int Immunopharmacol. (2012) 13:408–16. doi: 10.1016/j.intimp.2012.05.003, PMID: 22595192 PMC7106219

[45] Cunningham AL Lal H Kovac M Chlibek R Hwang SJ Diez-Domingo J. Efficacy of the herpes zoster subunit vaccine in adults 70 years of age or older. N Engl J Med. (2016) 375:1019–32. doi: 10.1056/NEJMoa1603800, PMID: 27626517

[46] Li X Zheng H Ma C Ji Y Wang X Sun D. Higher adjuvant radioactive iodine therapy dosage helps intermediate-risk papillary thyroid carcinoma patients achieve better therapeutic effect. Front Endocrinol (Lausanne). (2023) 14:1307325. doi: 10.3389/fendo.2023.1307325, PMID: 38298190 PMC10829775

[47] Sanchez-Martinez ZV Alpuche-Lazcano SP Stuible M Durocher Y. CHO cells for virus-like particle and subunit vaccine manufacturing. Vaccine. (2024) 42:2530–42. doi: 10.1016/j.vaccine.2024.03.034, PMID: 38503664

[48] Jie J Liu G Feng J Huo D Wu Y Yuan H. MF59 promoted the combination of cpG ODN1826 and MUC1-MBP vaccine-induced antitumor activity involved in the enhancement of DC maturation by prolonging the local retention time of antigen and down-regulating of IL-6/STAT3. Int J Mol Sci. (2022) 23:10887. doi: 10.3390/ijms231810887, PMID: 36142800 PMC9501507

[49] Stavnezer J. Immunoglobulin class switching. Curr Opin Immunol. (1996) 8:199–205. doi: 10.1016/S0952-7915(96)80058-6, PMID: 8725943

[50] Vidarsson G Dekkers G Rispens T. IgG subclasses and allotypes: from structure to effector functions. Front Immunol. (2014) 5:520. doi: 10.3389/fimmu.2014.00520, PMID: 25368619 PMC4202688

[51] Finkelman FD Holmes J Katona IM Urban JF Jr. Beckmann MP Park LS. Lymphokine control of *in vivo* immunoglobulin isotype selection. Annu Rev Immunol. (1990) 8:303–33. doi: 10.1146/annurev.iy.08.040190.001511, PMID: 1693082

[52] Klinman DM. IgG1 and IgG2a production by autoimmune B cells treated. Vitro IL-4 IFN-gamma. J Immunol. (1990) 144:2529–34. doi: 10.4049/jimmunol.144.7.2529 2108205

[53] Baird NL Zhu S Pearce CM Viejo-Borbolla A. C. Current In Vitro Models to Study Varicella Zoster Virus Latency and Reactivation. Viruses. (2019) 11:103. doi: 10.3390/v11020103, PMID: 30691086 PMC6409813

[54] Mangmee S Kardkarnklai S Phuphanitcharoenkun S Suthisawat S Li-Khit O Kamchompoo N. Characterization of neutralizing versus binding antibody and T cell responses to varicella-zoster virus in the elderly. Sci Rep. (2025) 15:13776. doi: 10.1038/s41598-025-98107-8, PMID: 40258885 PMC12012113

